# Analysis of *whiB7* in *Mycobacterium tuberculosis* reveals novel AT-hook deletion mutations

**DOI:** 10.1038/s41598-023-40152-2

**Published:** 2023-08-16

**Authors:** Olabisi Flora Davies-Bolorunduro, Bharkbhoom Jaemsai, Wuthiwat Ruangchai, Worakorn Phumiphanjarphak, Pakorn Aiewsakun, Prasit Palittapongarnpim

**Affiliations:** 1 Pornchai Matangkasombut Center for Microbial Genomics, Department of Microbiology, Faculty of Science, Mahidol University, Rama 6 Road, Bangkok, 10400 Thailand; 2 Department of Microbiology, Faculty of Science, Mahidol University, Rama 6 Road, Bangkok, 10400 Thailand; 3 Center for Tuberculosis Research, Microbiology Department, Nigerian Institute of Medical Research, 6 Edmund Crescent, P.M.B 2013, Yaba, 101012 Lagos Nigeria

**Keywords:** Computational biology and bioinformatics, Bacterial genes

## Abstract

Mutations in *whiB7* have been associated with both hypersusceptibility and resistance to various antibiotics in *Mycobacterium tuberculosis* (Mtb). Unlocking the secrets of antibiotic resistance in the bacterium, we examined mutations in the coding sequences of *whiB7* of over 40,000 diverse Mtb isolates. Our results unveil the dominant c.191delG (Gly64delG) mutation, present in all members of the lineage L1.2.2 and its impact on WhiB7's conserved GVWGG-motif, causing conformational changes and deletion of the C-terminal AT-hook. Excitingly, we discovered six unique mutations associated with partial or total deletion of the AT-hook, specific to certain sublineages. Our findings suggest the selective pressures driving these mutations, underlining the potential of genomics to advance our understanding of Mtb's antibiotic resistance. As tuberculosis remains a global health threat, our study offers valuable insights into the diverse nature and functional consequences of *whiB7* mutations, paving the way for the development of novel therapeutic interventions.

## Introduction

Tuberculosis (TB), is a severe bacterial disease that accounts for nearly 1.6 million human deaths and 10 million new cases worldwide annually^1,2^. The disease is caused by *Mycobacterium tuberculosis* (Mtb), consisting of nine major lineages (L1-9) with Lineages 1–4 (L1-4) distributed widely while the other lineages are more restricted to Africa^3,4^. Each lineage is further subdivided into several sublineages, which may have different epidemiological profiles.

The current treatment for TB involves a combination of several antibiotics; however, drug-resistant TB is a growing problem, with several hundred thousand cases reported annually^5,6^. One of the contributing factors to the drug resistance ability of Mtb is a transcription regulatory protein WhiB7. WhiB7 is one of the seven WhiB family of transcription regulators, specific to Actinobacteria. It interacts with the housekeeping sigma factor SigA, during transcription. WhiB7 contains a C-terminal motif called an AT-hook region (amino acid residues 80–91), which contains several positively charged amino acid residues, arginine and lysine, and binds to the AT-rich DNA regions upstream of transcription promoters of the regulated genes^7,8^, modulating their expression^9^. The SigA-WhiB7 interaction requires a triplet amino acid residue (EPW) motif adjacent to the conserved GVWGG motif, which forms a β-turn structure. The β-turn structure is located in a loop region and facilitate the protein–protein interaction^10^. WhiB7 is present across all non-pathogenic and pathogenic mycobacteria species including Mtb*, M. smegmatis,* and *M. abscessus*^11^. This protein has been linked to adaptive responses to antibiotic exposure and contributes to the intrinsic drug resistance of Mtb, playing part in multiple pathways including antibiotic export and chemical modifications of the antibiotics or their targets^12^. The protein has also been shown to be a redox-sensitive transcriptional regulator causing significant changes to thiol redox balance, which occurs shortly after antibiotic treatment^9,13,14^. The upregulation of *whiB7* transcription has been shown to be in response to various antibiotics of various structural classes including aminoglycosides, macrolides as well as metabolic signals elicited by these different antibiotics^9^.

There have been studies which suggested that mutations in *whiB7*, either in the coding sequence or its promoter region, can increase drug resistance. For instance, the CRYPTIC consortium reported that, in some Mtb variants predominant in Southeast Asia, some mutations in *whiB7* increased the minimum inhibitory concentration of ethionamide, suggesting that the mutations increase antibiotic-resistance to this drug^2^.

In contrast, there have been studies showing that disruption of *whiB7* promotes the sensitivity of Mtb to some antibiotics. For example, Warit et al*.*^13^ found that a frameshift deletion mutation at the nucleotide position 191 of the gene caused the bacteria to become hypersusceptible to the macrolide clarithromycin. Others reported that the same mutation caused hypersusceptibility to clarithromycin^15,16^, and showed that the *whiB7* LOF (loss of function) mutation could be found in the members of a specific sublineage of lineage 1. The L1.2.1 sublineage, described by Li et al.,^15^ as the mutant susceptible to clarithromycin, is equivalent to EAI2 defined by spoligotyping and has been recently renamed as L1.2.2,^17,18^ which will be used throughout this manuscript. It is predominant in Southeast Asia and accounts for 80% of TB cases in the Philippines^19^, and 10% in Thailand^20^, and has been subclassified into five sublineages including L1.2.2.2, equivalent to EAI2_NTB, mostly found in Thailand^17,19,21^. The other sublineages of L1.2.2 (L1.2.2.1, L1.2.2.3- L1.2.2.5) are equivalent to the spoligogroup EAI2_MNL. It should be noted that L1.2.1 is currently used to describe a newly discovered early branching sublineage of L1.2, isolated from patients in Europe, Papua, and Timor^19^. The finding that L1.2.2 is susceptible to clarithromycin opens an opportunity to explore the use of macrolides for the control of TB in the region. Establishing a connection between antibiotic susceptibility and lineage specificity is crucial because this prior information would help to direct the correct antibiotics in the treatment of Mtb of specific sublineages peculiar to different geographic locations.

Other studies which reported mutations in resistant genes associated with LOF and consequent susceptibility of Mtb variants that harbour the mutations include that of Walker et al.^22^ who characterised mutations within genes associated with resistance or susceptibility. Similarly, a recent study made an important contribution by identifying lineage-specific Mtb variants which have mutations on drug-resistant genes that confer hyper-susceptibility to varying antibiotics^23^.

Based on a large collection of whole genome sequences (WGS) of Mtb in the NCBI database, this study, aims to characterize the distribution of the mutations in *whiB7* across all Mtb lineages, with a particular focus on those in the coding sequences. The results from this study may provide insights into the consequences of these mutations, for explaining hypersusceptibility or resistance to antibiotics.

## Results

### Mutations in whiB7

A multiple sequence alignment of 40,520 *whiB7* coding sequences supplemented with its upstream and downstream sequences (200 bp on each side) was made to characterise a comprehensive set of *whiB7* variants. The dataset comprised sequences from a diverse range of Mtb isolates from 8 lineages across all continents. A total of 1235 alleles were identified, 1007 (51.5%) of which were singleton, i.e., found only in one isolate. On the other hand, we found that there was one allele shared by 89.2% of the total sequences analysed (36,125 sequences), which coincidentally was also the allele of reference H37Rv strain. As such, we named this variant as the wt allele and used this allele as the reference allele for *whiB7* mutation identification in this study. In our dataset, *whiB7* of all L7 and L8 isolates had the wt allele. The proportion of the wt allele was lowest in L1 at 68.3% (3,317/4,853), followed by L5 at 85% (136/162). More than 90% of the sequences of the L2, L3, L4, and L6 isolates were of the wt alleles (Supplementary Table S1).

**Figure 1:**
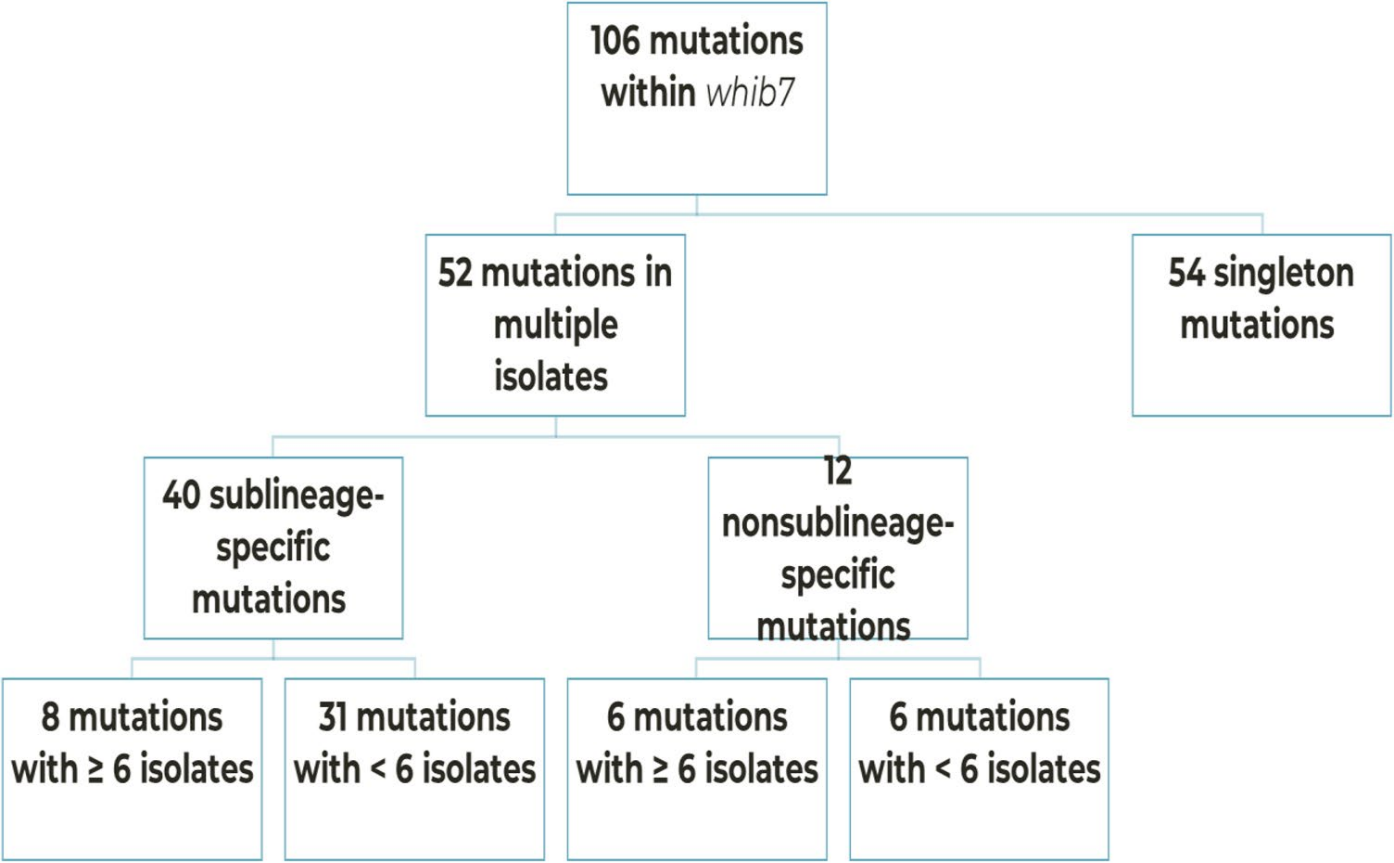
Distribution of mutations within the *whiB7.* Mutational landscape within the coding sequence of *whiB7*. A total of 106 mutations were identified, showcasing the diverse array of genetic alterations. About half of the mutations were singleton while the others occurred in more than one isolate. 40 of the latter were identified only in a single sublineage and referred to as sublineage-specific mutations. Our analysis was focused on mutations that occurred in 6 isolates or more.

The number of mutations observed in *whiB7* including the 200 bp of the flanking regions were 242. However, mutation analyses in this study focused on its coding sequence. One hundred and six mutations were identified in the coding sequence of *whiB7*, of which 90 were non-synonymous and 16 were synonymous. Seven nonsynonymous and one synonymous mutation were found within the AT-hook domain (Supplementary Table S2). Fifty-two of the 106 mutations were distributed across multiple isolates while 54 mutations were associated with singleton isolates. Forty of the 52 mutations, which occurred amongst multiple isolates, occurred specifically in a single sublineage (Fig. 1).

Eight of the group-specific mutations were identified in a minimum of 6 isolates including c.70G > C, c.91G > A, c.125 T > C, c.173A > G, c.177G > T, c.191delG, c.200G > T, (Supplementary Table S2). The eighth mutation comprised a set of 4Mutations (c.245_246insTT, 242G > A, c.237C > A, and c.238A > C) occurring in some members of the L2.2.AA3.1. Examination of these mutations revealed that they were combined to form a single replacement of an eight-nucleotide sequence 237-CAAGCGTC-244 by a seven-nucleotide sequence 237-GCATCTT, which is referred to as the 4M mutation in this study. Two of the mutations, 191delG and 4M, caused frameshifts. Among other group-specific mutations were missense mutations c.70G > C, c.91G > A, c.125 T > C, c.173A > G, and c.200G > T which occurred among some members of sublineages L4.7, L1.1.1.11, L2.2.AA3.1, L1.1.2.2^17,24^, and L5 respectively whereas c.177G > T was a silent mutation observed in L4.8.

**Figure 2:**
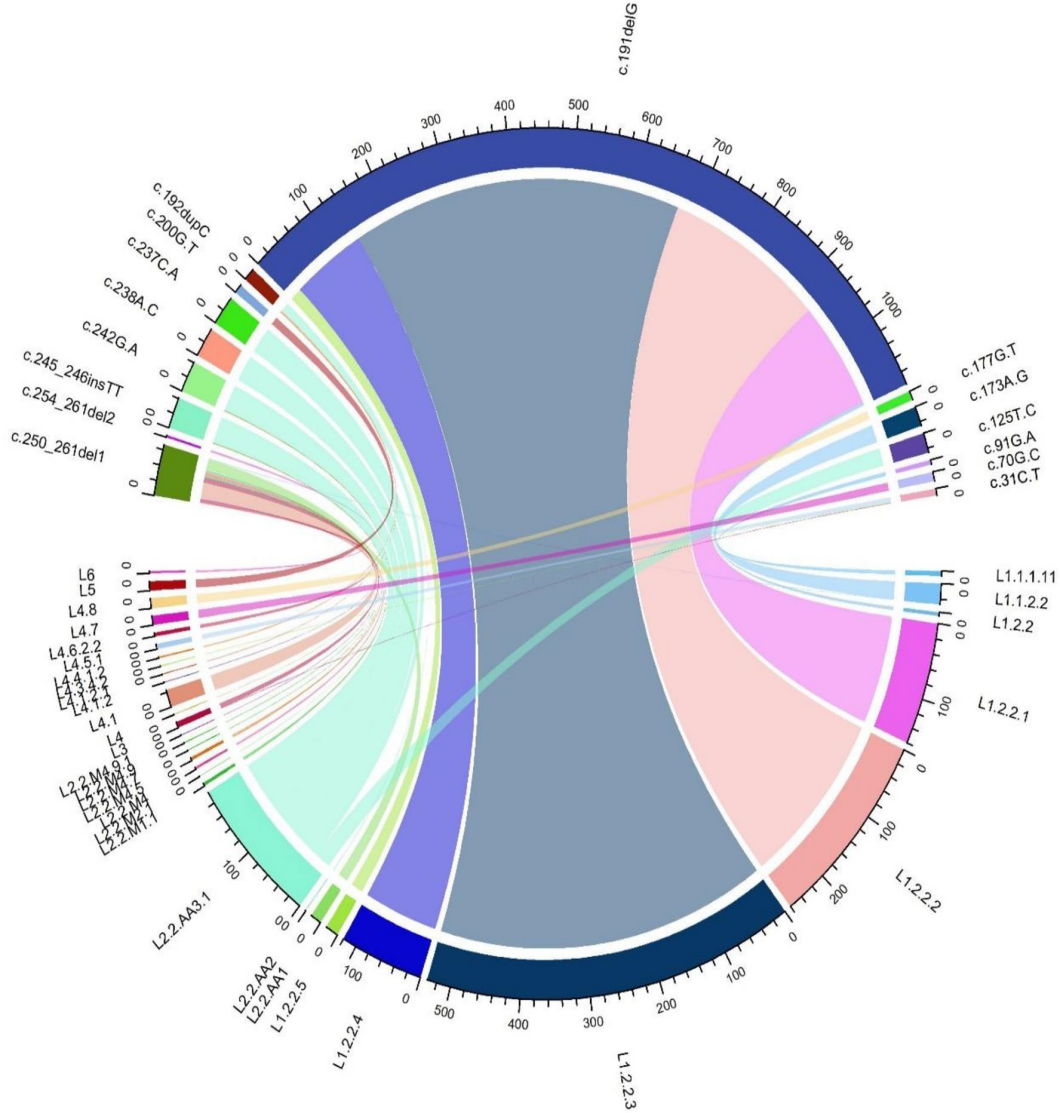
Lineage distribution of the abundant *whiB7* variants. The chord plot depicts some unique gene mutations on *whiB7* and some with homoplasy. Multicoloured chords show the association of mutations at various nucleotide positions of the genes (depicted with prefix c. + nucleotide position) with the different Mtb sublineages (L1-L6). The thickness of the coloured chords corresponds to the number of mutated isolates. The chord plots were drawn using the CirclizeR package in R version 4.2.1. c.250_261del1 refers to c.250_261delGGACGTCCGCGC and c.254_261del2 refers to c.254_261delGTCCGCGC.

The other 12 mutations including c.245_246insTT, 242G > A (parts of 4M) as well the long deletion 250_261GGACGTCCGCGC, occurred in more than one sublineage, thus indicating homoplasy. The sublineages which harboured these mutations were predominantly members of the L2 and L4 sublineages (Fig. 2).

We confirmed 191delG^13,15,16^ in the *whiB7* of 1098 isolates belonging to L1.2.2 (EAI2). There were only two isolates belonging to the L1.2.2.1 sublineage that did not harbour the mutation. The mutation accounted for 24.9% (1098/4395) of the *whiB7* mutated population. The mutation disrupted the conserved GVWGG motif and caused a frameshift that changed the downstream amino acid sequences and the loss of the AT-hook sequence. This deletion has been reported to cause hypersusceptibility of mutants to clarithromycin^13,15,16^. We did not detect the mutation among L1.2.1 isolates, which are sisters of L1.2.2 isolates, reported in Europe, Papua, and Timor. This indicated that the mutation occurred after the separation of L1.2.1 and L1.2.2 but before the basal diversification of L1.2.2. Another group-specific mutation (c.173A > G) affecting 2.2% (26/1157) of the L1.1.2.2 isolates was also observed.

**Figure 3:**
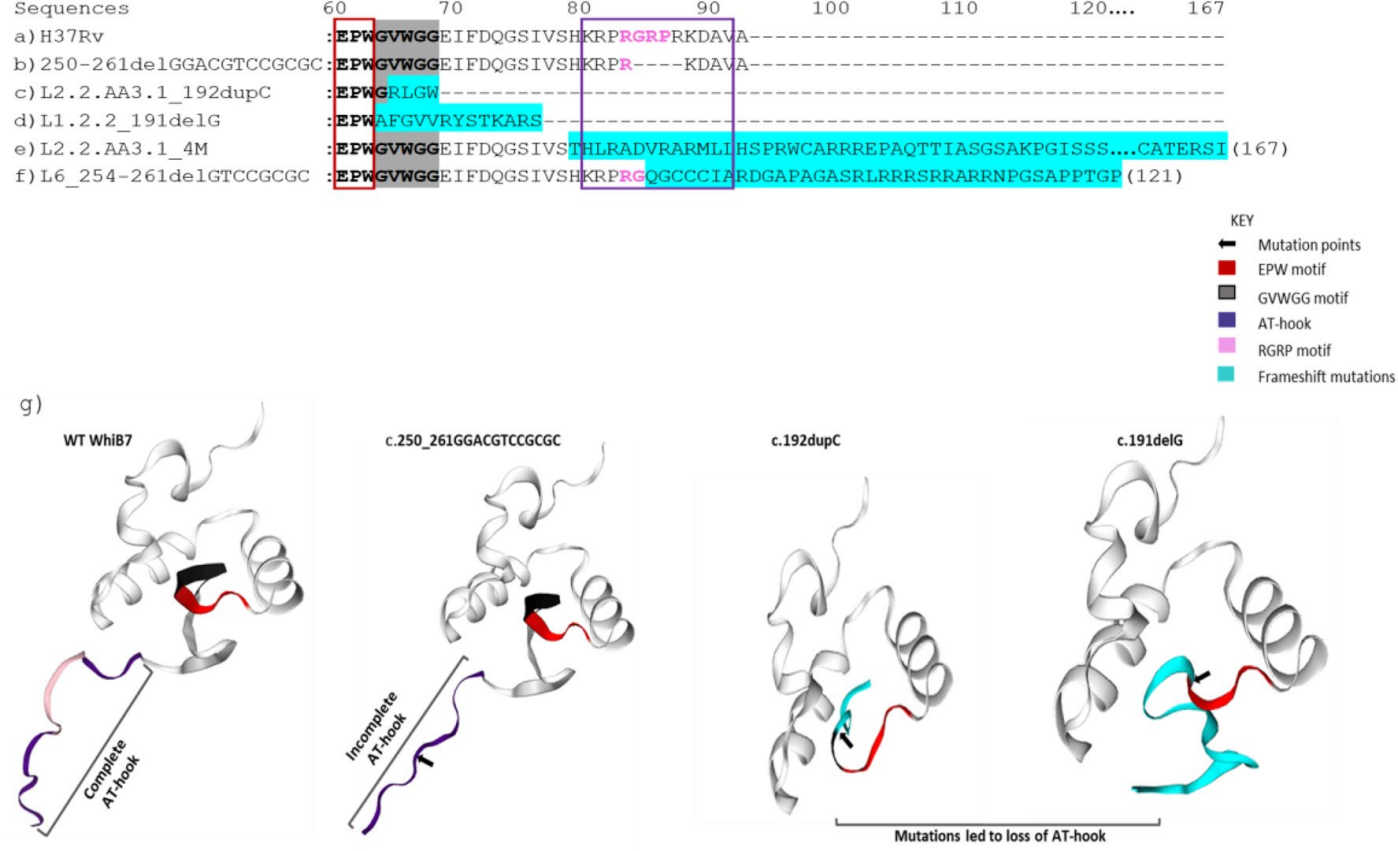
WhiB7 amino acid sequence alignment of the H37Rv sequence and various mutants with the affected AT-hook region. An alignment of some mutants highlighting the EPW conserved region, GVWGG motif, and the AT-hook region. The structure prediction was generated from Swiss-Model (https://swissmodel.expasy.org/) and subjected to quality assessment for accuracy and reliability of the predicted protein structure. The conserved EPW motif is highlighted with a red box. The β-turn GVWGG motif is shaded in grey with mutated residues shaded in blue. The ‘KRPRGRPRKDAV’ AT-hook region is highlighted with a blue box, with the core RGRP region in pink letters. (**a**) The wildtype sequence of WhiB7 in the H37Rv reference strain. (**b**) The mutation c.250_261delGGACGTCCGCGC, which occurred in 23 L4.1 isolates, 16 L2.2.AA1 isolates and 14 isolates in other sublineages. This mutation caused a deletion of 4 amino acid residues at the AT-hook region. (**c**) The c.192dupC mutation, which occurred in 13 L2.2.AA3.1 isolates resulting in a frameshift which led to the early termination of the protein at position 69. (**d**) The c.191delG mutation, which occurred in all L1.2.2 isolates, caused a frameshift and amino acid changes starting from position 64 and termination at position 77, resulting in the deletion of the entire AT-hook region. (**e**) The 4M mutation in 39 L2.2.AA3.1 isolates caused a frameshift and the elongation of the C terminal of the protein. (**f**) The other deletion at the AT-hook region, c.254_261delGTCCGCGC, which occurred in 3 L6 isolates and caused a frameshift and the elongation of the C terminal of the protein. (**g**) The protein structures of key variants showing the consistent presence of the EPW motif (red). The GVWGG motif (black) is disrupted in c.191delG and c.192dupC, resulting in complete deletions of the AT-hook region. The core region of the AT hook (RGRP, shown in pink) is absent in the c.250_261delGGACGTCCGCGC mutant.

The 4M mutation occurred in 39 isolates among 1029 isolates belonging to L2.2.AA3.1^24^. The frameshift caused by the mutation resulted in total amino acid residue changes from position 79 onwards (Fig. 3), including the sequence of the AT-hook region, and the delay of the translation termination to the amino acid position 167 instead of 92.

**Figure 4:**
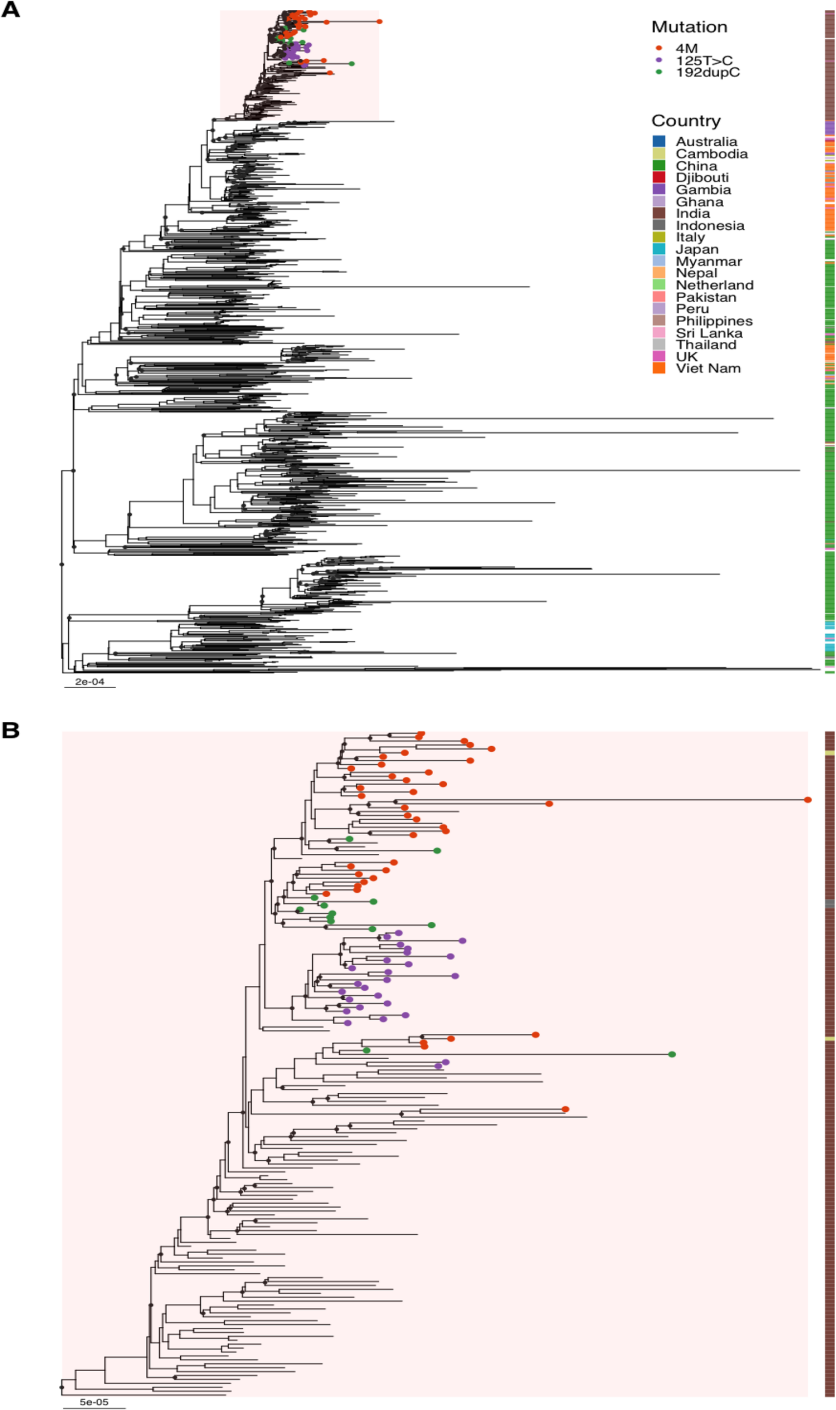
A phylogenetic tree of L2.2.AA3.1, showing the isolates with mutations: 4M (orange), c.192dupC (green), and c125T > C (purple). (**a**) Phylogenetic tree of all 1026 L2.2.AA3.1 isolates rooted with an L2.1 isolate. (**b**) Phylogenetic tree of the terminal 187 isolates of L2.2.AA3.1 shows isolates with the three mutations. The numbers of the isolates with mutations 4M, c.192dupC, and c.125 T > C were 39, 13, and 26 respectively. The tree was generated with IQ-TREE v2.1.3 and visualized with FigTree v1.4.4 and GGtree package in R Studio v4.2.1. The list of all the isolates in the dataset is in Supplementary Table S3.

The L2.2.AA3.1 isolates were primarily identified in China, India, and Vietnam, with the terminal clade exhibiting three distinct mutations as determined by phylogenetic analysis. Notably, 7.6% (78/1028) of the isolates bore one of the three mutations, i.e., 4M, c.192dupC or c.125 T > C, and were observed in four sister clades within the major terminal branch, with 48.1% (78/162) of these isolates originating from India (Fig. 4), with two 2 mutated isolates from Indonesia and two from Vietnam while the sources of the others were unknown. Most 4M and c.192dupC mutated isolates belonged to two terminal sister clades in the terminal branch while most c.125 T > C mutated isolates (24/26) formed another separate clade. All isolates of this clade were from India, as illustrated in Fig. 4. The c.192dupC mutation caused a frameshift and consequent amino acid residue changes starting from amino acid residue 65 as well as an early translation termination resulting in the truncation of the protein length from 92 to 68 amino acids and the loss of the AT-hook region.

The c.192dupC and c.125 T > C mutations were observed to be independently occurring in L4.4.1.2 and L4.1.2.1. Additionally, isolates belonging to L2.2.M1.1 and L2.2.M4.5 harboured a subset of the 4M mutation, c.245_246insTT, and c.242G > A (Supplementary Table S2).

### Mutation-induced changes to the AT-hook region of whiB7

Among relatively common mutations in *whiB7*, several lead to frameshifts and changes or loss of the amino acid sequences at the C-terminus, including the major mutation identified in sublineages L1.2.2 (c.191delG) and L2.2.AA3.1 (4M mutation and c.192dupC). The frameshifts generally occurred at positions before the AT-hook region of the gene, located at amino acid position 80 to 91 on its C-terminus^25^.

In addition, the mutations, c.250_261delGGACGTCCGCGC and c.254_261delGTCCGCGC directly affected the AT-hook region. The c.250_261delGGACGTCCGCGC mutation, occurred in 67 isolates belonging to 16 different sublineages, predominantly among L4.1 and L2.2.AA1, led to the loss of four amino acid residues from the AT-hook region, whereas the other mutation, 254_261delGTCCGCGC occurred only in some L6 isolates and caused the changes of the amino acid sequences, RGRP, from position 85 and elongation of the protein by 28 amino acids.

## Discussion

Mtb is a global health threat with multidrug-resistant (MDR) and extensively drug-resistant (XDR) strains posing a particular challenge for treatment^26^. Findings from many studies have established the associations between mutations in drug target genes in Mtb and drug susceptibility^27^. In this study, we identified mutations in the transcription regulator *whiB7* in 40,520 isolates of Mtb through the analysis of their WGS.

We confirm the presence of 191delG in a common albeit geographically localized sublineage L1.2.2 (EAI2), which are known to be associated with clarithromycin resistance^13,15,16^. The consequent sensitivity to clarithromycin may provide a better opportunity to treat MDR-TB patients caused by the sublineage with the antibiotic. It is worth noting that the prescription rate of clarithromycin for respiratory infections in Thailand is generally low, in comparison to other antibiotics^28^, minimizing the unintentional exposure of Mtb to clarithromycin. Nevertheless, its use in multidrug regimens for treating MDR-TB patients caused by L1.2.2 remains to be investigated. In Thailand, L1 (the Indo-Oceanic Lineage) and L2 (the East-Asian lineage) are equally predominant while L1.2.2 accounted for about a quarter of L1^21^. The rate of MDR cases was higher among L2 and the rate among L1.2.2 was less than 1% in northern Thailand^20^. Nevertheless, the burden and the MDR rate of L1.2.2 in the Philippines were much higher^19^. Globally the annual new cases of TB caused by the strains belonging to L1.2.2 was estimated to be around 600,000^17^, making exploring the use of clarithromycin worthwhile.

Evaluating the consequences of 191delG mutation revealed that it caused a dramatic change in the downstream amino acid sequence starting from the β-turn GVWGG motif, conserved among WhiB-like family of proteins^10^, resulting in the structural change and the complete loss of the AT-hook structure. The AT-hook structure is common among eukaryotic nuclear proteins but uncommon in bacteria. It is shown to enhance the binding of transcriptional regulatory complexes, comprising RNA polymerase-SigA holoenzyme, global regulators CarD and RbpA, and WhiB7, to the AT-rich DNA sequence motifs upstream of WhiB7-regulated promoters^29^. The role of these AT hook motifs, as well as the GVWGG-motif in conferring antibiotic resistance in vivo, has been established through targeted mutagenesis^30^. The study showed that different *whiB7* mutants without the GVWGG-motif and AT hook region did not restore the wt resistance typically exhibited by intact WhiB7 thus revealing the importance of the AT-hook DNA binding domain as a requirement for optimal WhiB7 function. Therefore, these described consequences of 191delG suggest a molecular mechanism on how the mutation, contributing to the hypersusceptibility of L1.2.2 to clarithromycin naturally occurs.

The fact that almost all isolates of this genotype harbor the same mutation suggests that this mutation occurred in the early ancestor of L1.2.2. However, it is not clear whether the mutation has provided any selective advantages or contributed to the success or widespread of the sublineage, as increased sensitivity to an antibiotic by itself would not increase survival. However, WhiB7 is related to the expression of about 100 genes and transcriptional re-wiring of stress-response pathways can enhance tolerance to antibiotics^31^ or probably other environmental stress. Moreover, the occurrence of collateral sensitivity, where resistance to certain antibiotics may influence susceptibility to other antibiotics, has also been described^31–33^, suggesting that a reverse phenomenon may be possible.

The frameshift mutations with LOF effects, are not unique to L1.2.2 but also affect small proportions of other sublineages. Further observations have unveiled several mutations that interfere with the functionality of the AT-hook of WhiB7, either partially or completely. Our research has identified a few mutations in some L2.2.AA3.1 isolates that triggered frameshifts ahead of the AT-hook domain, such as the 4M mutation and c192dupC. Both mutations cause frameshifts and consequently the total loss of the AT-hook. c192dupC also disrupts the GVWGG motif. Unfortunately, the sensitivity to clarithromycin of the mutants was unknown.

Phylogenetic analysis revealed that the basal isolates of L2.2.AA3.1 were primarily identified in China^24^, whereas the mutants located in the terminal clade, which contained the 4M mutation, were predominantly found in India. This hints that the origin of the mutation occured after the sublineage was transferred from China.

There were also other mutations that affect the AT-hook directly. The c.250_261delGGACGTCCGCGC mutation caused the deletion of the core AT-hook sequence motif, RGRP, essential for tethering the protein to the DNA^31^. Interestingly the mutation was identified in 17 sublineages. The homoplasy suggested a positive selection of the RGRP loss in some situations. A homoplastic signal of selection pressure has been described for many drug resistance mutations^34,35^. c.254_261delGTCCGCGC, identified only in a few L6 isolates, also affects the RGRP motif and causes downstream frameshift, leaving only the first few amino acid residues of the AT-hook intact. Interestingly a recent study also revealed the presence of another mutation, R85C, affecting the RGRP motif of 25 MTB isolates in India^36^.

The mutations in *whiB7* have various physiologic effects. It induces antibiotic resistance in Mtb against several antibiotics, including kanamycin and streptomycin, the second-line drugs for TB^29^. An earlier study by the Cryptic consortium^2^ used oligopeptide probes to link elevated minimum inhibitory concentration (MIC) of ethionamide to several substitutions within the AT-hook region. WhiB7 also plays an important role in cellular redox homeostasis. In *Streptomyces*, SigR, a sigma factor that is involved in the cellular response to oxidative stress, is controlled by WblC, an ortholog of Mtb WhiB7. In Mtb, the homologs of SigR, SigE and SigH, are also likely to be controlled by WhiB7, which aids the response to oxidative stress by activating genes that help the bacteria survive and restore their redox balance^10^.

*whiB7* is regulated by VapC21, the toxin component of a toxin-antitoxin system VapBC21^28^. The VapBC toxin-antitoxin systems are differentially expressed in stress conditions, which Mtb may encounter during infection. Recently the SenX-RegX3 two-component system was also found to regulate the expression of *whiB7* in response to phosphate starvation, acid stress, and hypoxia^37^. WhiB7 itself is autoregulated and is involved in regulating *eis* (enhanced intracellular survival, an N-acetyl transferase), *tap* (a multidrug transporter) and *erm* (ribosomal methyltransferase)^12^*.* The loss of transcription of *erm* by the loss of AT hook in the c191delG mutant is likely to contribute to the unusual sensitivity of L1.2.2. to clarithromycin^38^. It is possible that other mutations that cause the loss of AT hook region also lead to the sensitivity to clarithromycin.

While our study is limited by the lack of targeted gene knock-out experiments to confirm the causal relationship between LOF in the *whiB7*, and clarithromycin sensitivity, our results provide a starting point for further research into the role of this gene in TB drug resistance. A better understanding of the mechanism of WhiB7 and its AT hook region will be critical for the development of novel drugs targeting WhiB7. The availability of several natural mutations in WhiB7 will also provide an opportunity to study the roles of the transcription regulator in the physiology, survival, and pathogenesis of Mtb. Nevertheless, the scope of biochemical roles of WhiB7 is still unclear and the full implications of the mutations remain to be discovered.

Overall, the mutations in *whiB7*, which affect its AT-hook region, may cause both hypersusceptibility and resistance to different TB antibiotics. c.191delG causes the loss of both the β-turn GVWGG motif and the AT-hook region. There are other natural mutations that may affect only the AT-hook core motif (RGRP), the entire AT-hook region, or additionally the β-turn GVWGG motif. Further investigations are required to discern the exact effects of these mutations and their implications for Mtb antibiotic resistance.

## Methods

### Sample acquisition and sequencing

We downloaded 40,520 raw whole-genome sequencing (WGS) short read data from the NCBI Sequence Read Archive (SRA) (https://www.ncbi.nlm.nih.gov/sra/) and the European Nucleotide Archive (ENA), which were mostly generated using the Illumina platform (https://www.ebi.ac.uk/ena). The samples were obtained from diverse geographic regions worldwide.

### Data preprocessing and variant calling

We trimmed the raw sequencing reads to remove low-quality reads using Trimmomatic v0.39^39^ with the following parameters: sliding window trimming with a window size of 4 and a read quality threshold of 30. The trimmed reads were then aligned to the H37Rv reference genome (NC_000962.3) using BWA-MEM v0.7.17^40^. Picard's MarkDuplicates was used to identify and remove duplicate reads (https://github.com/broadinstitute/picard). Per-sample variant calling was performed using GATK HaplotypeCaller v3.8^41^ with a haploid model, excluding bases with a quality score below 20. We used GATK GenotypeGVCFs to generate a single variant call format (VCF) file containing the variants in the *whiB7* gene^20^.

### SNV annotation and phylogenetic reconstruction

We annotated the SNVs using SnpEff v4.3t^42^ with the H37Rv reference genome. A VCF file was utilized to present genomic variants, which encompassed variant information from all samples and stored the variant data in a singular file. Multiple sequence alignment was performed using Aliview v1.17.1^43^. We reconstructed the phylogenetic tree for the lineages using IQ-TREE v2.1.3^44^ with ultrafast bootstrap supports from 1000 replications. The best-fit nucleotide substitution model was determined using ModelFinder v1.7.1^45^. We used lineage 2.1 as an outgroup for rooting the tree of L2.2.AA3.1 isolates and tree visualization was performed using FigTree v1.4.4 and GGtree package in R Studio v4.2.1.

### Protein structure homology modeling

We performed protein structure homology modeling of the WhiB7 alleles^46,47^. The amino acid sequence of the protein which contained the mutations were used to generate the model structure using ProMod3^48^, a comparative modelling engine based on OpenStructure^49^. Structure prediction was done with the SWISS-MODEL platform (https://swissmodel.expasy.org/) which incorporates known structures and algorithms to predict the protein's structure based on its sequence and subsequently generating a model of the protein. The generated model was then subjected to quality assessment and validation using scores from QMEANDisCo^50^ and QMEAN Z^51^, ensuring the reliability of the predicted protein structure.

### Mutation distribution among lineages

We constructed a chord plot to determine the association of the mutations and corresponding lineages using the Circlize package in RStudio v4.2.1.

### Supplementary Information

Supplementary Table S1.

Supplementary Table S2.

Supplementary Table S3.

## Data Availability

All data pertaining to the manuscript have been provided in the form of figures and tables. The sequence data are available in the NCBI Sequence Read Archive (SRA) (https://www.ncbi.nlm.nih.gov/sra/) and the European Nucleotide Archive (ENA) (https://www.ebi.ac.uk/ena). Supporting information is available as Supplementary Tables S1-S3.
