# Supplementary material for: Analysis of whiB7 in Mycobacterium tuberculosis reveals novel AT-hook deletion mutations
Source: Sci Rep. 2023 Aug 16;13:13324. doi: 10.1038/s41598-023-40152-2 (PMC10432532; doi:10.1038/s41598-023-40152-2)
Supplement: Supplementary file 1 — Supplementary Table S1. [file 41598_2023_40152_MOESM1_ESM.pdf]

Supplementary Information for the manuscript

Analysis of *whiB7* gene in *Mycobacterium tuberculosis* reveals novel AT-hook deletion mutations

Olabisi Flora Davies-Bolorunduro[1,2,3], Bharkbhoom Jaemsai[2], Wuthiwat Ruangchai[1], Worakorn Phumiphanjarphak[2], Pakorn Aiewsakun[1,2], Prasit Palittapongarnpim[1,2*]

[1]Pornchai Matangkasombut Center for Microbial Genomics, Department of Microbiology, Faculty of Science, Mahidol University, Rama 6 Road, Bangkok 10400 Thailand

[2]Department of Microbiology, Faculty of Science, Mahidol University, Rama 6 Road, Bangkok 10400 Thailand

[3]Center for Tuberculosis Research, Microbiology Department, Nigerian Institute of Medical Research, 6 Edmund Crescent, P.M.B 2013, Yaba 101012, Lagos – Nigeria [*]Corresponding author: Prasit Palittapongarnpim,

Pornchai Matangkasombut Center for Microbial Genomics, Faculty of Science, Mahidol University, Rama 6 Road, Bangkok 10400 Thailand.

Email: prasit.pal@mahidol.ac.th

Supplementary Table S1. The number of isolates with or without mutations in *whiB7* by lineages.

Lineage	Total Number of isolates	Number of isolates with the wt allele	No of isolates with mutant Alleles	Isolates with wt alleles (%)	Isolates with mutant alleles (%)
L1	4853	3317	1536	68	32
L2	14205	13226	979	93	7
L3	4648	4283	365	92	8
L4	16081	14627	1453	91	9
L5	162	136	26	84	16
L6	540	505	35	94	6
L7	30	30	0	100	0
L8	1	1	0	100	0
**Total**	**40520**	**36125**	**4394**	**89**	**11**

Supplementary Table S2. List of the mutations in the *whiB7* originally derived from the annotation of SNVs using SnpEff v4.3t with the H37Rv reference genome (NC_000962.3). The table is in an Excel file, Supplementary Table 2. The table presents variant information for each mutation, including the number of isolates with mutation, mutation in *whiB7* and nucleotide change, amino acid change, predicted genetic effects of mutations, predicted effects on gene functions, loss of function, and sublineages that the mutations were identified. The mutations were identified using SnpEff v4.3t 35 with the H37Rv as a reference.

Supplementary Table S3. List of the 40,520 Mtb isolates. The table is in an Excel file, Supplementary Table S3. The table lists the accession numbers of the sequence data available in the NCBI Sequence Read Archive (SRA) (https://www.ncbi.nlm.nih.gov/sra/) and the European Nucleotide Archive (ENA) (https://www.ebi.ac.uk/ena), used in this study.
